# Optimization of chromium and tannic acid bioremediation by *Aspergillus niveus* using Plackett–Burman design and response surface methodology

**DOI:** 10.1186/s13568-017-0504-0

**Published:** 2017-11-14

**Authors:** Prachi Chaudhary, Vinod Chhokar, Pragati Choudhary, Anil Kumar, Vikas Beniwal

**Affiliations:** 1 Department of Biotechnology, Maharishi Markandeshwar University, Mullana, Ambala 133207 India; 2 Department of Bio & Nano Technology, Guru Jambheshwar University of Science & Technology, Hisar, Haryana 125001 India

**Keywords:** Tannery effluent, *A. niveus*, Tannic acid, Chromium, Plackett–Burman, RSM

## Abstract

A chromium and tannic acid resistance fungal strain was isolated from tannery effluent, and identified as *Aspergillus niveus* MCC 1318 based on its rDNA gene sequence. The MIC (minimum inhibitory concentration) of the isolate against chromium and tannic acid was found to be 200 ppm and 5% respectively. Optimization of physiochemical parameters for biosorption of chromium and tannic acid degradation was carried out by Plackett–Burman design followed by response surface methodology (RSM). The maximum chromium removal and tannic acid degradation was found to be 92 and 68% respectively by *A. niveus*. Chromium removal and tannic acid degradation was increased up to 11 and 6% respectively after optimization. Scanning electron microscopy (SEM) and Fourier transform infrared spectroscopy (FTIR) was used to investigate biosorption phenomena.

**Electronic supplementary material:**

The online version of this article (10.1186/s13568-017-0504-0) contains supplementary material, which is available to authorized users.

## Introduction

Water is the important natural resource for all living forms. This natural resource is being polluted by rapid growth of population, metropolitanization and mechanization (Singanan et al. 2007). Industrialization leads to several environmental problems like water, land and air pollution. An extensive volume of wastewater originated from industries which are released into channels either untreated or inadequately treated causing water pollution.

The wide spread use of chromium in various products and application in different industrial processes has emitted considerable environmental contamination (Sultan and Hasnain 2007). Tanning industries widely used chromium compounds to convert animal skins and hides into leather, mainly chromium sulphate. Tanneries produced waste water in range of 30–35 L/kg with total Chromium 23.3–42.5 mg/L. Other than tanning industry, metal finishing, petroleum refining, iron and steel production, inorganic chemicals production, textile manufacturing, and pulp-producing industries also contributed in chromium pollution. Cr (chromium) waste is liberated into the environment via deprived storage, leakage or improper treatment and disposal practices. In India, more than 50% of the total chromium effluent discharge originates from the leather, iron and steel industries (Garg et al. 2012, 2015). Human when exposed to Cr(VI) (hexavalent chromium) they may suffer from several health hazards such as allergic dermatitis, nasal irritation, renal tubular necrosis, eardrum perforation ulceration, skin irritation, lung carcinoma, epidermal dermatitis and increase risk of cytotoxic and genotoxic effects (cell death, cell transformation and gene mutation) and respiratory tract cancer (Camargo et al. 2005). Accumulation of Cr takes place mainly in spleen, liver, kidneys, and bone marrow. At high levels Cr damage cell membranes, disrupt cellular function, modify enzyme specificity, and damage structure of DNA (Farag and Zaki 2010; Smrithi and Usha 2012). It has adverse impacts on aquatic species as it penetrates into cell walls, accumulates in fish tissues. The pollution potential of the effluents from the tannery industry produces phytotoxic effects and high accumulation of heavy metals resulting in stress for plants. Trivalent chromium (Cr(III)) is 10–100 times less toxic than hexavalent chromium, the reason is that trivalent chromium complexes are impermeable through cellular membranes (Lu and Yang 1995).

Tannins are water soluble polymeric polyphenols having different molecular weight. Their molecular weight ranges from 500 to 3000 g/mol. Tannins have astringency property i.e. they precipitate phospholipids in cell membrane because of this cell wall becomes impermeable from solutions that increases its stability to water, bacteria and heat. Vegetable tanning is the oldest process used in the leather industries. Vegetable tannins are divided in two categories: hydrolysable tannins and condensed tannins. Hydrolysable tannins are esters of gallic acid (galotannins) or ellagic acid and are hydrolysed by acids or enzymes into monomers. Hydrolysable tannins (HT) cause necrosis of the liver, hemorrhagic gastroenteritis, jaundice, kidney damage with proximal tubular necrosis, photosensitization and death in severe cases (Dollahite et al. 1962; Murugan 2010). Condensed tannins are flavonoid monomers (Filippich et al. 1991; Bhat et al. 1998; Jadhav et al. 2011). The poisonous effects of Condensed tannins are less well known; in general CT reduces the digestive capacity. The tannin rich effluent is dark coloured because of the presence of tannins and its perseverance for a long time, unless microbial oxidation takes place. Tannins readily sink in the water bodies and reduce the availability of oxygen in deeper water layers. Tannins in irrigation water, makes it unfit and decrease the yield of crops. It inhibits the biodegradative enzymes and soil microorganisms and slow down the rate of decomposition of soil organic matter (Hernandez et al. 2005; Mahadevan and Muthukumar 1980). Biodegradation of condensed tannins is poorly understood due to its chemical complexity and reactivity. However, biodegradation of hydrolysable tannins produces important molecules like gallic acid and ellagic acid which shows a wide range of biological activities such as antimicrobial, antiviral, antioxidant and anticancer etc. (Chavez-Gonzalez et al. 2014; Choubey et al. 2015).

A number of researchers have reported the removal of chromium from the wastewater, but there is little information on tannic acid removal from the tannery wastewater. However, there is no report on the multiple toxicant removal. The present work aims to isolate indigenous predominant adapted fungal strains from tannery effluents which possess the ability to detoxify and degrade Cr and tannic acid from tannery effluent.

## Materials and methods

### Isolation of fungal strain

Effluent sample was collected from leather industry located at Karnal, Haryana, India. Samples of wastewater were taken from inlet of the treatment plant. Samples were mixed to get a composite sample and stored at 4 °C (Mahmood et al. 2013). The isolates were isolated using enrichment culture technique from tannery effluent samples (Sharma and Adholeya 2012). Chromium resistant and tannic acid degrading fungi were isolated separately. 10 mL effluent was enriched in 100 mL potato dextrose broth (PDB, pH 5.5) amended with 50 mg/L (50 ppm) filter sterilized K_2_Cr_2_O_7_ (Potassium dichromate) as Cr(VI) source and 0.1% filter sterilized tannic acid (as described by Chhokar et al. 2010), followed by incubation at 30 °C for 120 h at 120 rpm. After incubation, resistant fungi were isolated by serial dilution followed by spread plate technique on potato dextrose agar plates amended with 50 mg/L (50 ppm) Cr(VI) and 1 mg/mL (0.1%) tannic acid. The strain was sub-cultured at an interval of 4–5 weeks and routinely maintained on Potato Dextrose Agar (PDA) slants under refrigerated conditions.

A fungal spore inoculum was prepared by adding 2.5 mL of sterile distilled water containing 0.1% Tween 80 to a fully sporulated culture. The spores were dislodged using a sterile inoculation loop under strict aseptic conditions, and the number of spores in the suspension was counted using a Neubauer chamber. Finally, 1 mL (0.5 mL of each strain) of the prepared spore suspension was used as the inoculum, with a concentration of 5 × 10^9^ spores (Beniwal et al. 2015).

### Screening of potential strain

Screening was carried out for chromium removal and tannic acid degradation potentiality of fungal isolates under minimal salt medium (K_2_HPO_4_ 0.5 g/L, KH_2_PO_4_ 0.5 g/L, MgSO_4_ 0.5 g/L, NH_4_Cl 1.0 g/L, Glucose 0.5 g/L, pH 5.5). Different concentrations of chromium (100, 150 and 200 ppm) and tannic acid (0.1, 0.25, 0.5%) were amended in the medium along with effluent and inoculated with fungal isolates. Flasks were incubated at 30 °C, 120 rpm for 120 h. On the basis of atomic absorption spectroscopy and tannic acid assay potential strains were selected. Selected isolates were further screened out simultaneously for tolerance against Cr(VI) and tannic acid.

### Identification of the isolated fungal strain

Total genomic DNA of the isolate was extracted according to the method of Wu et al. (2009). 18S rRNA gene fragment was amplified by PCR using the set of primers NS1 (5-GTAGTCATATGCTTGTCTC-3) and NS4 (5-CTTCCGTCAATTCCTTTAAG-3) designed to anneal to conserved regions of the fungi’s 18S rRNA. A 15-μL reaction volume included 1 μL (50 ng/μL) template, 7.5 μL 2× Taq PCR MasterMix, 5.9 μL ddH2O, and 0.3 μL of each primer (10 μmol/μL), performed in a Peltier Thermal cycler (M. J. Research, Inc, Japan) according to the following conditions: 35 cycles of 94 °C for 30 s (de-naturation), primer annealing at 55 °C for 50 s, and 72 °C for 150 s, and a final extension at 72 °C for 10 min. PCR products were then electrophoresed on 1.5% agarose gels using 1× TAE buffer (40 mM Tris acetate; 1 mM EDTA; pH 8.0), containing 200 ng/mL ethidium bromide to detect the product. Images were captured on a Syngene Bioimaging system (Syngene, U.K.). Sequencing of PCR products were conducted by excising the bands from the agarose and purifying the complementary DNA (cDNA) using AuPrep GelX kit (Life Technologies Ltd.). The purified cDNA was sequenced. The resulting sequences were analyzed and aligned using Blast Local Alignment Tool (BLAST) program at the National Centre for Biotechnology Information (NCBI).

### Selection of significant variables by Plackett–Burman design

Plackett–Burman design containing eleven variables was selected to study their effect on chromium bioremidiation and tannic acid removal. Selected variables along their symbol code and range are shown in Additional file 1: Table S1. Among the nutrients, Glucose, NH_4_Cl, MgSO_4_, K_2_HPO_4_, chromium and tannic acid were selected. Furthermore, the physical parameters pH, inoculums size, incubation period, temperature and agitation speed were tested. For each assay, the chromium removal and residual tannic acid was measured and calculated in terms of percentage (%). The software Design Expert 9.0 (Stat Ease. Inc.^®^, US) was used to analyze the experimental data.

### Optimization by response surface methodology and statistical design

Box–Behnken is a response surface design, requires three level factorials, coded as − 1, 0, and + 1. Box–Behnken model generate designs with desirable statistical properties but, with only a fraction of the experiments required for a three-level factorial, the quadratic model is suitable for it. The coefficients of the quadratic model may be calculated using standard regression techniques (Francis et al. 2003). In Box–Behnken design, number of experiments is calculated by following equation:

1 $$ {\text{N}} = {\text{k}}_{ 2} + {\text{k}} + {\text{cp}} $$

where k is the factor number and cp is the replicate number of the central point (Edrissi et al. 2008). The statistical software package ‘Design Expert 6.0’, Stat-Ease, Inc., Minneapolis, USA was used to analyze the experimental design.

The obtained data of chromium removal, and residual tannic acid was subjected to analysis of variance (ANOVA), suitable to the design of experiments. The mathematical relationship of the independent variables and the responses were calculated by the second order polynomial equation i.e.

2 $$ \begin{aligned} Y = \beta_{0  } + \beta_{1} A + \beta_{2} B + \beta_{3} C + \beta_{4} D + \beta_{11} A^{2 } + \beta_{22} B^{2 } + \beta_{33} C^{2 } + \beta_{44} D^{2 } + \beta_{12} AB \hfill \\ \, + \beta_{13} AC + \beta_{14} AD + \beta_{23} BC + \beta_{24} BD + \beta_{34} CD \hfill \\ \end{aligned} $$

where Y = predicted response; β_0_ = intercept; β_1_, β_2_, β_3_, β_4_ = linear coefficients; β_11_, β_22_, β_33_, β_44_ = squared coefficients; β_12_, β_13_, β_14_, β_23_, β_24_, β_34_ = interaction coefficients and A, B, C, D are coded value of variables to determine the optimum level for maximal degradation of tannic acid 3D graphs were produced to get to know the effect of selected variables individually and in combination (Beniwal et al. 2015).

### Tannic acid assay and analysis of chromium

Tannic acid was measured by the method of Hagerman and Butler (Hagerman and Butler 1989). Briefly 3 mL of triethanolamine (1%, v/v) was added to 1 mL of filtered culture, followed by addition of 1 mL FeCl_3_ (0.01 M FeCl_3_ in 0.01 N HCl) solution. The mixture was kept for 15 min at room temperature for color stabilization. Color was read at 530 nm against the blank by UV–Vis spectrophotometer, Model-2450 (Shimadzu, Japan).

Total chromium was estimated by atomic absorption spectrophotometer (SensAA GBC).

### Characterization of Cr(VI) biosorption on the basis of surface studies

#### Fourier transforms infrared (FTIR) spectroscopy

FTIR spectrum study was carried out to explain the change in the functionalities of the microbial culture in the presence of chromium. The spectra were collected using Shimadzu equipped with diffuse reflectance accessory with the range of 400–4000 cm^−1^ (Tunali et al. 2006). The biomass was harvested by filtering the culture. The 4 h lyophilized sample was grounded in a pestle and mortar with KBr. The background obtained from KBr disc was automatically took away from the sample discs. The spectra were composed using Perkin Elmer BX II system.

#### Scanning electron microscopy

Morphological changes resulting from the metal stress was examined by Scanning Electron Microscopy (SEM) (JSM-6510 OVL, Japan). Culture was centrifuged at 6000 rpm for 15 min. The supernatant was discarded and fungal beads pellet was washed 3–4 times with 0.1 M phosphate buffer (pH 7.2). After that fungal pellet was fixed in 2.5% glutaraldehyde and dehydrated with 30–90% ethanol. Final dehydration in 100% ethanol was carried out for 8–10 min and dried to remove moisture. Chromium treated and untreated (control) samples were coated with 90 Å thick gold under vacuum to increase the electron conduction and to improve the quality of the micrographs. Coated cells were viewed at 15 kV with scanning electron microscopy (Michalak et al. 2014).

## Results

### Isolation, screening and identification of fungal strain

Fifteen chromium resistance and tannic acid degrading fungal strains were isolated from the tannery effluent samples by continuous transfer on minimum salt medium amended with 50 ppm filter sterilized K_2_Cr_2_O_7_ as Cr(VI) and 0.1% tannic acid as the sole carbon source. The isolates Cr 5 was selected among all the isolated strains for their fast growth rate and relatively higher resistance toward chromium and tannic acid. The MIC of the strain was found to be 200 ppm and 1% for chromium and tannic acid respectively. During simultaneous screening Cr(VI) and tannic acid removal was found around 81 and 62% respectively.

Colonies of Cr 5 on agar plate were dense, thin margin, conidial areas white with pale yellowish shade at the centre, reverse greyed-orange. The amplified partial 18S rRNA gene was sequenced and compared with similar information available at the GenBank by an online alignment search. The BLASTn of the isolate was showing 98% homology with *Aspergillus niveus* (KM613137.1) suggesting that the isolate is highly likely to be *A. niveus.* The sequence was submitted to GenBank with an accession number of KX129954. The strain has been deposited to Microbial Culture Collection, National Centre for Cell Science, India with a reference number MCC 1318.

### Selection of significant variables by Plackett–Burman design

Table 1 Shows the Plackett–Burman experimental design of *A. niveus* and results obtained from experiments. The adequacy of the model was calculated and the variables showing statically significant effects were screened on the basis of % contribution, coefficient estimate and P value. The variables with significant effects were those with a P < 0.05, selected for further optimization study. Extensive deviation was found in Cr(VI) reduction (59.3–99.9%) and residual TA (12.4–92.0%), which reflects the importance of medium optimization to accomplish high reduction. Cr concentration, with a probability value of 0.0043 and 0.033, TA concentration (0.0061, 0.0232), glucose (0.0032, 0.0421), and NH_4_Cl (0.0042, 0.0151) were found to have positive influence on removal and selected for further optimization studies (Table 2). The lower probability values indicate the more significant factors for Cr and tannic acid removal. Agitation speed exerted a negative effect on the responses. Including agitation speed all other insignificant variables were neglected and the optimum levels of the four variables, (Cr, TA, glucose and NH_4_Cl) were further determined by an RSM design. Rest of the variables were worked best at their middle value.

**Table 1:** Plackett Burman experimental design matrix of eleven variables

Run	X_1_	X_2_	X_3_	X_4_	X_5_	X_6_	X_7_	X_8_	X_9_	X_10_	X_11_	% Cr removal	% residual TA
1	5	30	96	110	3	100	2.75	0.5	1	0.5	0.5	90.75	54.06
2	7	20	144	180	1	150	5	0.8	0.5	0.2	0.2	74.83	74.73
3	3	40	144	40	5	150	5	0.2	0.5	0.2	0.8	91.44	92.03
4	3	40	144	180	1	50	0.5	0.8	0.5	0.8	0.8	59.32	12.49
5	3	20	48	180	1	150	5	0.2	1.5	0.8	0.8	88.71	92.05
6	7	40	48	40	1	150	0.5	0.8	1.5	0.2	0.8	99.57	70.8
7	7	20	144	180	5	50	0.5	0.2	1.5	0.2	0.8	97.63	27.24
8	7	40	144	40	1	50	5	0.2	1.5	0.8	0.2	97.21	74.73
9	3	40	48	180	5	50	5	0.8	1.5	0.2	0.2	72.31	74.73
10	3	20	144	40	5	150	0.5	0.8	1.5	0.8	0.2	94.15	55.95
11	3	20	48	40	1	50	0.5	0.2	0.5	0.2	0.2	87.11	40.41
12	7	20	48	40	5	50	5	0.8	0.5	0.8	0.8	60.16	74.73
13	7	40	48	180	5	150	0.5	0.2	0.5	0.8	0.2	99.99	90.22

**Table 2:** Screening of critical factors for *A. niveus*

Chromium					Tannic acid			
Variable	Stdized effect	% contribution	Coefficient estimate	P value	Stdized effect	% contribution	Coefficient estimate	P value
pH	6.06	4.53	3.03	0.0089	7.47	2.23	3.73	0.4348
Temperature	2.88	1.02	1.44	0.0187	8.32	2.77	4.16	0.4016
Incubation period	1.12	0.16	0.56	0.0479	− 17.63	12.45	− 8.81	0.2112
Agitation speed	− 6.14	4.66	− 3.07	0.0088	− 6.20	1.54	− 3.10	0.4935
Inoculum size	1.49	0.27	0.74	0.0361	8.28	2.75	4.14	0.4028
Cr concentration	12.49	19.27	6.25	0.0043	28.58	32.70	14.29	0.0333
TA concentration	8.85	9.68	4.43	0.0061	30.98	38.44	15.49	0.0232
Glucose	16.96	35.51	8.48	0.0032	8.87	13.15	4.44	0.0421
NH_4_Cl	12.79	20.19	6.39	0.0042	1.82	0.13	0.91	0.0151
MgSO4	3.89	1.87	1.95	0.0138	3.37	0.46	1.69	0.6774
K_2_HPO_4_	4.80	2.84	2.40	0.0112	6.91	1.91	3.45	0.4593

### Response surface methodology

The experimental design was aimed to identify the best levels of the selected variables, i.e. NH_4_Cl (0.5–1.5 g/L), glucose (0.2–0.8 g/L), Cr concentration (50–200 ppm), and tannic acid concentration (5–50 ppm) (Additional file 1: Table S2). Box Behnken experimental design of *A. niveus* with actual and predicted values was presented in Table 3. The second-order polynomial equation was used to find out the relationship between variables and response. The regression equation coefficients were calculated and data was fitted to a second-order polynomial equation.

**Table 3:** Box–Behnken experimental design of *A. niveus*

Run	A: glucose g/L	B: tannic acid %	C: metal dose ppm	D: NH_4_Cl g/L	Cr removal %		Residual TA %	
					A* value	P* value	A* value	P* value
1	0	+ 1	− 1	0	80.97	82.39	49.03	49.10
2	0	+ 1	0	+ 1	86.11	85.54	49	48.94
3	− 1	− 1	0	0	89.42	90.50	4.38	4.38
4	0	0	+1	− 1	89.98	90.13	26.32	26.37
5	+1	0	0	+ 1	84.21	84.50	26.29	26.28
6	0	0	0	0	89.84	88.66	26.3	26.26
7	+ 1	0	− 1	0	83.66	83.55	26.33	26.31
8	− 1	0	− 1	0	83.81	82.91	26.59	26.55
9	0	0	− 1	+ 1	83.26	83.21	26.47	26.45
10	+ 1	0	0	− 1	–		–	
11	+ 1	+ 1	0	0	89.54	88.57	48.84	48.88
12	0	0	0	0	90.61	88.66	26.26	26.26
13	0	0–1	0	+ 1	88.85	89.25	4.47	4.39
14	0	0	+ 1	+ 1	85.67	85.06	26.27	26.37
15	0	0	0	0	85.76	88.66	26.3	26.26
16	0	0	− 1	− 1	78.12	78.82	26.54	26.48
17	0	0	0	0	89.14	88.66	26.28	26.26
18	+ 1	− 1	0	0	90.62	91.16	4.29	4.34
19	+ 1	0	+ 1	0	89.40	89.67	26.45	26.39
20	− 1	0	0	+ 1	88.13	88.69	26.4	26.47
21	0	+ 1	0	− 1	88.51	87.48	48.98	48.96
22	0	− 1	− 1	0	86.73	85.68	4.26	4.34
23	0	− 1	+1	0	91.94	91.08	4.45	4.45
24	− 1	0	+1	0	90.48	89.95	26.34	26.26
25	− 1	+1	0	0	89.31	88.88	48.97	48.95
26	0	0	0	0	87.91	88.66	26.17	26.26
27	− 1	0	0	− 1	84.38	84.66	26.28	26.35
28	00	− 1	0	− 1	88.06	87.99	4.45	4.41
29	00	+1	+1	0	88.54	90.15	48.81	48.80

A* value actual value, P* value predicted value

### Model validation

**Fig. 1:**
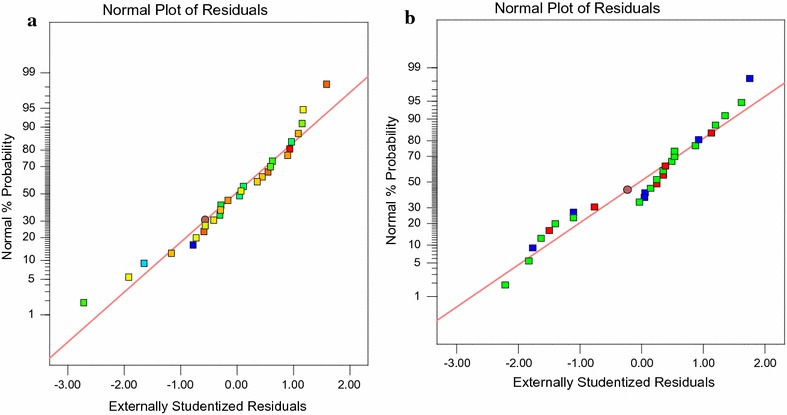
Normal plot of studentized residuals verses normal % probability of *A. niveus* for bearing the experiment for **a** Cr(VI) and **b** residual tannic acid

The acceptability of the model and fitness was estimated by ANOVA (analysis of variance) and regression coefficients for the experimental design used. The ANOVA of *A. niveus* (Table 4) for the quadratic model of residual tannic acid and Cr removal indicated the F value of 7275.21 and 8.73 respectively with a very low probability value (Pmodel > F = 0.0001, 0.0002 respectively) indicates significant level at 95% confidence interval. At the same time, relatively lower value of coefficient of variation (CV%) = 1.67 and 0.29 for Cr removal and residual tannic acid (TA) indicated a better precision and reliability of the experiments carried out. The determination coefficient (R^2^) of the model (tannic acid; 0.90 and Cr removal 1.0) showed the good agreement between the experimental results and the theoretical values predicted by the model and it showed that the model was appropriate to represent the real relationship among the selected factors (Table 5). The insignificant lack of fit test also pointed out that the model was suitable to navigate the design space. The residuals were used to check the homogeneous variance assumption by plotting the (studentized) residuals against the predicted probability values. Normal probability plot of the residuals nearly followed a straight line that indicates a normal distribution of residuals (Fig. 1a, b). The final predictive equation was as follows:

3 $$ \begin{aligned} {\text{Cr removal }} = \, + \, 8 8. 6 6 { } + \, 0.0 8 9 { }*{\text{ A }} - 1.0 5 { }*{\text{ B }} + { 3}. 2 9 { }*{\text{ C }} - \, 0. 1 7 { }*{\text{ D }} - \, 0. 2 4 { }*{\text{ AB }} - 0. 2 3 { }*{\text{ AC}} \hfill \\ \, - { 2}. 1 8 { }*{\text{ AD }} + \, 0. 5 9 { }*{\text{ BC }} - \, 0. 80 \, *{\text{ BD }} - { 2}. 3 6 { }*{\text{ CD }} + \, 0. 1 6 { }*{\text{ A}}^{ 2} \hfill \\ + \, 0. 9 6 {\text{ B}}^{ 2} - 2. 2 9 { }*{\text{ C}}^{ 2} - { 2}.0 6 { }*{\text{ D}}^{ 2} \hfill \\ \end{aligned} $$

4 $$ \begin{aligned} {\text{Residual TA }} = \, +\, 2 6. 2 6 { } - \, 0.0 2 8 { }*{\text{ A }} + { 22}. 2 8 { }*{\text{ B }} - \, 0.0 4 8 { }*{\text{ C }} - { 8}.0 8 3 {\text{ E }} \hfill \\ {-} \, \,00 3 { }*{\text{ D }} - 1.000{\text{ E }}{-} \, 00 2 { }*{\text{ AB }} + \, 0.0 9 3 { }*{\text{ AC }} - 0.0 6 4 { }*{\text{ AD }} - \, 0. 10 \, *{\text{ BC }} \hfill \\ + \, 0.000 \, *{\text{ BD }} + { 5}.000{\text{ E }} - 00 3 { }*{\text{ CD }}0.0 4 1 { }*{\text{ A}}^{ 2} + \, 0. 3 3 { }*{\text{ B}}^{ 2} + \, 0.0 7 5 { }*{\text{ C}}^{ 2} + \, 0.0 80{\text{ D}}^{ 2} \hfill \\ \end{aligned} $$

**Table 4:** Analysis of variance (ANOVA) of *A. niveus* for the fitted quadratic polynomial model for residual tannic acid and chromium

Response variable	Source	Sum of squares	Degree of freedom	Mean square	F value	P value prob > F
Tannic acid	Model	5956.30	14	425.45	72757.21	< 0.0001^a^
	Lack of fit	0.064	9	7.149E−003	2.45	0.2016^b^
	Pure error	0.012	4	2.920E−003		
	Cor total	5956.38	27			
Chromium	Model	259.18	14	18.51	8.73	< 0.0002^a^
	Lack of fit	13.13	9	1.46	0.40	0.8804^b^
	Pure error	14.42	4	3.60		
	Cor total	286.73	27			

^a^Significant

^b^Non significant

**Table 5:** Statistical significance of residual tannic acid and chromium

Model terms	Chromium	Tannic acid
Std. dev.	1.46	0.076
Mean	87.25	26.48
%CV	1.67	0.29
PRESS	107.81	0.46
R-squared	0.9039	1.00
Adj R-squared	0.8004	1.00
Pred R-squared	0.6240	0.9999
Adeq precision	11.580	799.719

### Interactive effects of two variables

**Fig. 2:**
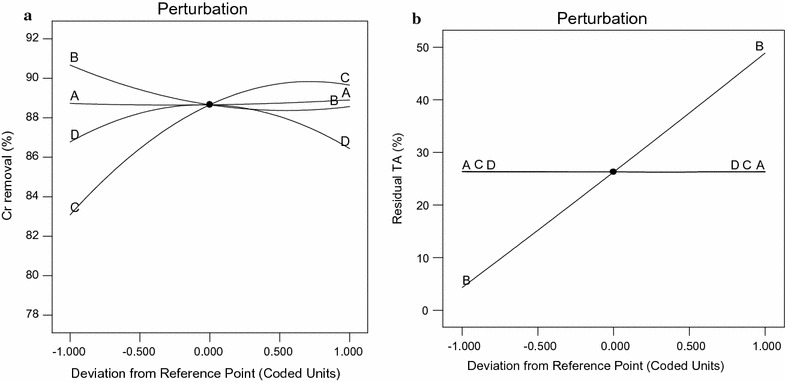
Overlay plot of perturbation of *A. niveus* for **a** Cr(VI) and **b** Residual tannic acid. A—glucose, B—tannic acid, C—metal dose and D—NH_4_Cl

**Fig. 3:**
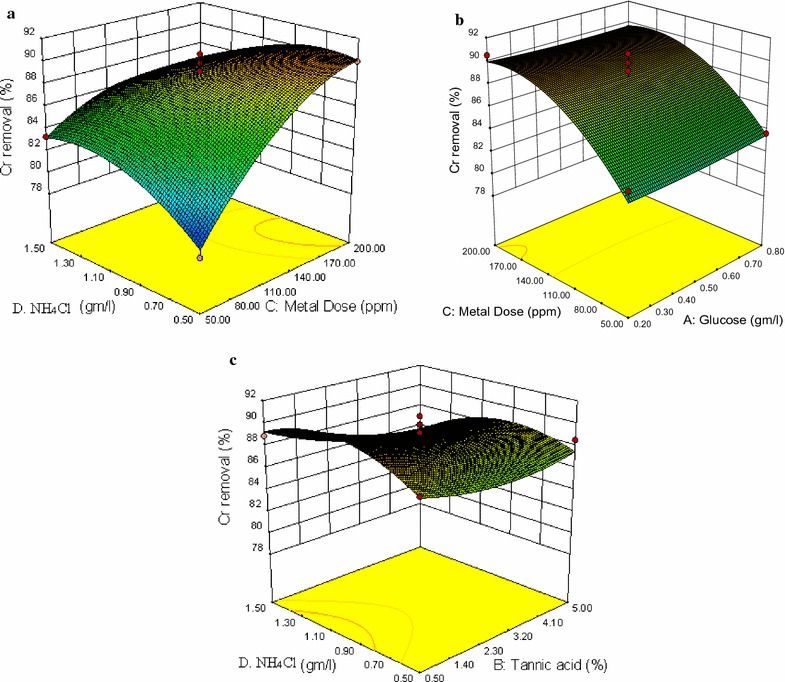
Three dimensional plots of *A. niveus*. **a** Three-dimensional plot of residual Cr(VI) as a function of metal dose and glucose at constant tannic acid (2.75%) and NH_4_Cl (1 g/L). **b** Three-dimensional plot of residual Cr(VI) as a function of metal dose and tannic acid at constant glucose (0.5 g/L) and NH_4_Cl (1 g/L). **c** Three-dimensional plot of residual Cr(VI) as a function of NH_4_Cl and metal dose at constant glucose (0.5 g/L) and tannic acid (2.75%)

The compassion of the response to the two interacting variables was represented by the three dimensional graphs by holding the other variable at the central values. On the basis of quadratic polynomial Eqs. (3 and 4) of the response surface methodology. The effect of each variable was further estimated by the use of perturbation plots to show how the response changes as each variable moves from the chosen reference point, with all other factors held at constant reference values (Liong and Shah 2005). Figure 2a shows the perturbation plot of Cr removal. Although all variable showed significant quadratic effects, the curve with the most prominent change was the perturbation curve of NH_4_Cl followed by metal dose (Chromium concentration). Glucose and tannic acid was found to be the least prominent variable compared to the other variables. The response surface curves are plotted to explain the interaction of the variables and to determine the optimum level of each variable to reach a maximum response. Figure 3a clearly demonstrates that Cr removal was sensitive even to small changes in NH_4_Cl concentration with a maximum response at 1.10 g/L. Cr removal increases with increase of metal dose. Optimum metal dose was recorded at 200 ppm (Fig. 3a). The interactive influence of NH_4_Cl–tannic acid and metal dose–glucose on Cr removal is illustrated in Fig. 3b, c. Three dimensional response surface plots of *A. niveus* demonstrated that tannic acid and glucose had no significant effect on Cr removal.

**Fig. 4:**
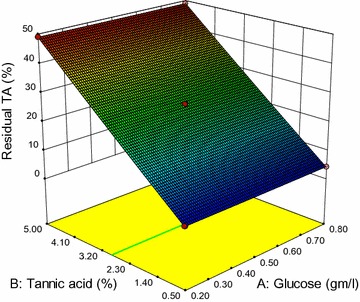
Three-dimensional plot of residual tannic acid as a function of tannic acid and glucose at constant NH_4_Cl (1.0 g/L) and metal dose (125 ppm)

Figure 2b showed that only tannic acid concentration showed significant effect on residual tannic acid. However, the other three factors did not have any significant role. Figure 4 shows that the residual tannic acid increases with an increase in TA concentration. However, other factors did not show any significant effect on the response (Fig. 4).

### Characterization of Cr(VI) biosorption on the basis of surface studies

The evaluation of morphological changes in response to Cr biosorption in *A. niveus* was performed by scanning electron microscopy (SEM). It was observed that after incubation of 96 h, the hyphae were cylindrical, septate, and branched. However in the presence of 180 mg/l of chromium, there was complete disruption and disintegration of mycelium in comparison to control (Additional file 1: Figure S1).

The FTIR spectra (400–4000 cm^−1^) of Cr(VI) loaded *A. niveus* was studied to confirm the presence of functional groups that are usually responsible for the biosorption process (Additional file 1: Figure S2). The frequencies of the spectrum bands and their assignments are listed in Additional file 1: Table S3. Peak frequencies in between 3429 and 3435 cm^−1^ attributed to –OH of glucose and –NH stretching of the protein and acetamide group. Changes were also detected in the absorption peak shifted at 1639 cm^−1^ corresponding to amide I (protein C–O stretching). A shift in the wave number 2367 cm^−1^ was due to the –CH stretching of methyl and methylene groups. A peak in between 617 and 669 cm^−1^ was the result of –CH bending of alkenes.

## Discussion

A total number of 15 fungi were isolated from tannery effluent samples. Cr 5, identified as *A. niveus* was found to be the most efficient isolate. Shugaba et al. (2010) isolated *A. niger* and *A. parasiticus* from the tannery sludge sample. Srivastava and Thakur (2006) reported *Aspergillus* sp. from tannery effluent. Das and Santra (2008) isolated *A. flavus* from Kolkata tannery effluent.

Cr, TA, glucose and NH_4_Cl were selected among 11 variables by using Plackett–Burman design. Generally, lower probability value (less than 0.05) of a parameter indicated higher significance of that parameter. A positive coefficient indicated that the higher concentrations of the variable is best for increasing Cr(VI) removal and residual tannic acid, whereas negative values indicated the vice versa (Pulimi et al. 2012). Mohan et al. (2013); Costa Souza et al. (2015) and Melo et al. (2014) found TA significant for tannase production by *Aspergillus* sp. in Plackett–Burman study. Mabrouk et al. (2014) studied Cr reduction by *Halomonas* sp. M-Cr by applying Plackett–Burman study and found that glucose significantly affects Cr removal in tannery effluent.

Mohan et al. (2014) also observed that with increase of metal dose Cr removal increases in *A. flavus*. Masood and Malik (2011) also find out that chromium adsorption was increased by *Bacillus* sp. FM1 with increasing metal concentration. This may be due to the interaction and binding affinity of chromium with sequestering sites of the organism increase with increased chromium concentration. Metal accumulation by fungi increases with increasing in initial metal ion concentration. This may be possibly due to increase electrostatic interactions of metal ions on the cell surface. Under suitable growth medium *Aspergillus* sp. produce spherical mycelia which help in metal accumulation (Prasenjit and Sumathi 2005). Karaca et al. (2010) reported that maximum Pb biosorption was occurred at pH 5 by *A. niveus.*

The capability of microorganisms to degrade tannins has been featured to the construction of tannase, an important enzyme able of catalyzing gallotannins to gallic acid and glucose (Ilori et al. 2007; Beniwal et al. 2013). In *Bacillus sphaericus*, with the increase of tannic acid tannase production increases i.e. tannic acid degradation increases (Raghuwanshi et al. 2011). *A. niger* strains were able to grow in a high concentration of tannic acid (10%). The highest tannase production by *A. niger* was occurred at 5% tannic acid and response surface plots suggested that even more tannase production could be obtained when increasing the tannic acid concentration. The results of the present study are in accordance with the previous report of Rodriguez-Duran et al. (2011). Sharma et al. (2007) also obtained the maximum tannic acid degradation at 5% tannic acid by *A. niger*. Tannins have a property to form complexes with fungal or bacterial exozymes, this is how tannins slow down the biodegradation process. Tannins also form complexes with metals (Field and Lettinga 1992). Tannic acid has chelating property, so it is possible that chromium was chelated by tannic acid in the medium. Ammonium chloride did not show any effect on residual tannic acid. This is probably due to the absorption of inorganic ions and the possibility of formation of complexes between tannins and protein structures. Therefore, significant variables were subsequently evaluated (Paranthaman et al. 2009; Mondal and Pati 2000). Venil et al. (2011) reported maximum removal at the minimum levels of NH_4_Cl in *Bacillus* sp.

Scanning electron microscopy (SEM) and Fourier transform infrared spectroscopy (FTIR) was used to investigate adsorption phenomena. A disruption and disintegration of mycelium was found in SEM (Additional file 1: Figure S1). This was probably due to the precipitation of Cr within the matrix of mycelium of fungi (Verma et al. 2011).

Peak frequencies in between 3429 and 3435 cm^−1^ attributed to –OH of glucose and –NH stretching of the protein and acetamide group. Similar observations were made by Tunali et al. (2005) in *Neurospora crassa*. Manasi and Lolly (2014) also reported the presence of –OH, –CH, C–O at their respective wavelengths of 3422, 1210 and 2926 cm^−1^ in *A. niger.* Khambhaty et al. (2009) reported the participation of –OH (hydroxyl group), –CH_2_ (alkane group), –NH_3_ (amino group) and phosphorous groups (–P) for Cr(VI)) binding to *Aspergillus* sp. The FTIR spectra showed the presence of ionizable functional groups (i.e., carboxyl, hydroxyl, phosphate and amino groups, methyl and methylene) that are able to interact with metal ions (Sethuraman and Balasubramanian 2010).

The present study indicates bioremediation potential of *A. niveus* against Cr and tannic acid degradation in tannery effluent. The maximum Cr(VI) removal and tannic acid degradation was found to be 92 and 68% respectively at 1.10 g/L NH_4_Cl, 200 ppm Cr concentration, 0.2 g/L glucose and at 5% TA concentration by *A. niveus*. Cr(VI) removal and tannic acid degradation was increased up to 11 and 6% respectively after optimization. Hernandez et al. reported 67% tannic acid degradation by *A. niger*. Shugaba et al. (2010) reported chromium removal and tannic acid degradation by the immobilized beads of spores of *A. niger* and *A. paraciticus* and found 97% chromium removal. Sharma and Adholeya (2012) reported 99% chromium removal by *A. lentulus*. Mishra and Malik (2012) reported 71% Cr removal by *A. lentulus*. Srivastava and Thakur (2006) observed 70% chromium removal by *Aspergillus* sp. SEM and FTIR were used to investigate biosorption of chromium by *A. niveus*. According to these observations *A. niveus* can be used in treatment of chromium and tannic acid rich industrial effluent. The microbe based technology for the removal of metals from the waste water is a cost-effective and easy to use process and has great potential for future applications.

Up to best of our knowledge *A. niveus* is firstly isolated from tannery effluent and there is no report available on *A. niveus* for chromium removal and tannic acid degradation. However one report on Pb removal is available (Karaca et al. 2010).

## Additional file

**Additional file 1.** Additional tables and figures.

## Abbreviations

RSM: response surface methodology
ANOVA: analysis of variance
SEM: scanning electron microscopy
FTIR: Fourier transform infrared spectroscopy
TA: tannic acid
