# Supplementary material for: Optimization of chromium and tannic acid bioremediation by Aspergillus niveus using Plackett–Burman design and response surface methodology
Source: AMB Express. 2017 Nov 14;7:201. doi: 10.1186/s13568-017-0504-0 (PMC5686038; doi:10.1186/s13568-017-0504-0)
Supplement: Supplementary file 1 — Additional file 1. Additional tables and figures. [file 13568_2017_504_MOESM1_ESM.pdf]

AMB EXPRESS

Optimization of chromium and tannic acid bioremediation by *Aspergillus niveus* using Plackett – Burman Design and Response Surface Methodology

Prachi Chaudhary[1], Vikas Beniwal[1*], Pragati Choudhari[2], Anil Kumar[2] and Vinod Chhokar[2]

[1]Department of Biotechnology, Maharishi Markandeshwar University, Mullana-133207, Ambala, India

[2]Department of Bio & Nano Technology, Guru Jambheshwar University of Science & Technology, Hisar-125001, Haryana, India.

*__Corresponding author:__

Vikas Beniwal, [1]Department of Biotechnology, Maharishi Markandeshwar University, Mullana-133207, Ambala, India.

Phone No. +919416768062

Fax No. +911731274375

Email: beniwalvikash@gmail.com

Table S1: Variables and coded levels used for Plackett – Burman study.

Variables	Symbols	Coded levels -1	Coded levels +1
pH	X_1	3	7
Temperature (^oC)	X_2	20	40
Incubation (h)	X_3	48	144
Agitation speed (rpm)	X_4	40	180
Inoculum (%)	X_5	1	5
Chromium concentration (ppm)	X_6	50	150
Tannic acid concentration (%)	X_7	0.5	5
Glucose (gm/l)	X_8	0.2	0.8
NH_4Cl (gm/l)	X_9	0.5	1.5
$MgSo_4$ (gm/l)	X_{10}	0.2	0.8
K_2HPo_4 (gm/l)	X_{11}	0.2	0.8

Table S2: Levels of independent variables (factors) used in RSM.

	Variables	Symbols	Coded levels		
			**-1**	**0**	**+1**
A. niveus	NH_4Cl (gm/l)	A	0.5	1.0	1.5
	Metal dose (chromium) (ppm)	B	50	125	200
	Tannic acid concentration (%)	C	0.5	2.75	5.0
	Glucose (gm/l)	D	0.2	0.5	0.8

Table S3: IR absorption bands and corresponding possible groups of *A. niveus* before and after treatment of Cr.

**Wavenumbers (cm^{-1})**		
**Before treatment**	**After treatment**	**Functional groups**
1639	1648	-OH, C-O (stretching due to proteins)
1053	1033	Phosphate functional groups (P-OH, P=O)
617	669	Alkene (-CH bending)
2367	2344	CH (stretch), methyl and methylene group
3435	3429	Hydrogen-bonded O–H stretching and N–H stretching of secondary amides

Figure Captions

Fig S1. Scanning electron micrograph of *A. niveus* (a) control (10 kV, x2500, 10 micron) (b) Cr loaded (10 kV, x2500, 10 micron).

Fig S2. FTIR spectra of *A. niveus* **(a)** control **(b)** Cr (VI) loaded.

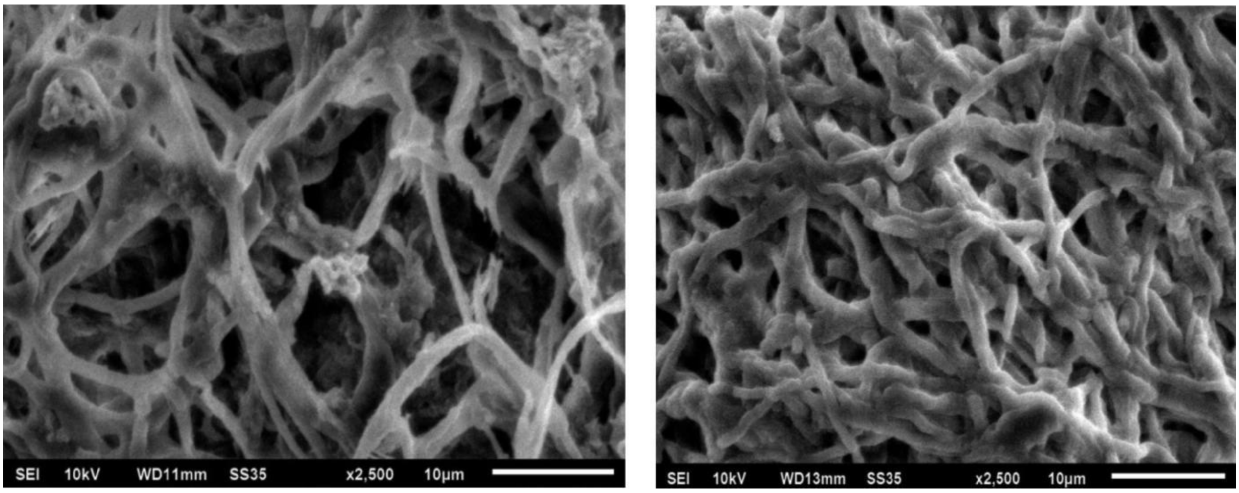

Fig S1: Scanning electron micrograph of *A. niveus* (a) control (10 kV, x2500, 10 micron) (b) Cr loaded (10 kV, x2500, 10 micron).

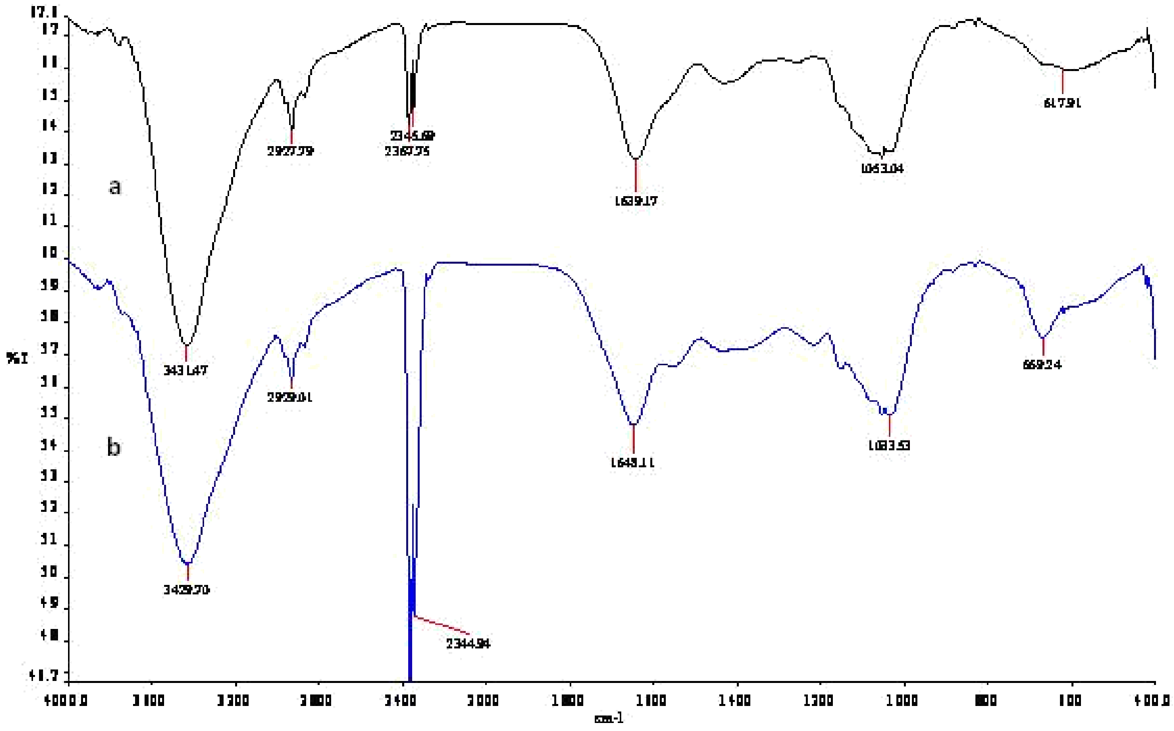

Fig S2: FTIR spectra of *A. niveus* **(a)** control **(b)** Cr (VI) loaded.
